# Melatonin targets mitochondrial trifunctional enzyme HADHA to improve lipid metabolism in metabolic dysfunction-associated steatotic liver disease

**DOI:** 10.1186/s43556-026-00461-0

**Published:** 2026-05-12

**Authors:** Yongping Zhu, Yanqing Liu, Rui Liu, Junzhe Zhang, Yuqing Meng, Li Liu, Xin Liu, Dandan Liu, Liwei Gu, Linying Zhong, Xianyu Xu, Ying Li, Jinyan Xu, Lingyun Dai, Shengnan Shen, Jigang Wang

**Affiliations:** 1 State Key Laboratory for Quality Ensurance and Sustainable Use of Dao-di Herbs, Artemisinin Research Center, and Institute of Chinese Materia Medica, China Academy of Chinese Medical Sciences, Beijing, 100700 China; 2 College of Animal Science and Technology, Henan Agricultural University, Zhengzhou, 450002 China; 3 Department of Critical Care Medicine, Guangdong Provincial Clinical Research Center for Geriatrics, Shenzhen Clinical Research Center for Geriatrics, Shenzhen People’s Hospital (The First Affiliated Hospital, Southern University of Science and Technology; The Second Clinical Medical College, Jinan University), Shenzhen, 518020 China; 4 Guangdong Pharmaceutical University, Guangzhou, 510006 China; 5 School of Pharmacy, Chengdu University of Traditional Chinese Medicine, Chengdu, 611137 China; 6 School of Pharmacy, Jiangxi University of Traditional Chinese Medicine, Nanchang, 330004 China; 7 Department of Pharmacological Sciences, Yong Loo Lin School of Medicine, National University of Singapore, Singapore, 117600 Singapore

**Keywords:** MASLD, Melatonin, HADHA, CETSA, Lipid metabolism

## Abstract

Metabolic dysfunction-associated steatotic liver disease (MASLD) is a clinicopathological syndrome characterized by abnormal accumulation of fat within hepatocytes, and there is currently no standardized clinical treatment available. Consequently, there is an urgent need to discover new pharmacological interventions and to investigate novel therapeutic targets for MASLD. Melatonin, known for its multifaceted biological functions, has shown therapeutic potential for the treatment of MASLD. However, the underlying mechanisms remain unclear, particularly since the direct targets of melatonin remain poorly understood. In our study, we found that melatonin significantly improved various indicators in a mouse MASLD model and protected palmitic acid-induced mouse hepatocytes from lipid accumulation. We successfully identified and validated the mitochondrial trifunctional enzyme α-subunit HADHA as a binding target for melatonin using the cellular thermal shift assay (CETSA). This interaction enhanced the expression of peroxisome proliferator-activated receptor gamma coactivator 1-alpha (PGC-1α), which subsequently promotes mitochondrial biogenesis and accelerates lipid metabolism. In addition, melatonin reduced lipid accumulation and ameliorated MASLD through its regulatory effect on key proteins involved in fatty acid metabolism, including acyl coenzyme A oxidase 1 (ACOX1), cluster of differentiation 36 (CD36), and fatty acid synthase (FASN). Importantly, these beneficial effects were diminished when HADHA was knocked down. In conclusion, our study suggests that melatonin ameliorates MASLD through HADHA-mediated regulation of mitochondrial biogenesis and lipid oxidation, highlighting its potential as a promising therapeutic agent for MASLD. These results offer novel insights into the role of melatonin in the treatment of MASLD.

**Supplementary Information:**

The online version contains supplementary material available at 10.1186/s43556-026-00461-0.

## Introduction

Metabolic dysfunction-associated steatotic liver disease (MASLD), previously known as nonalcoholic fatty liver disease (NAFLD), is a prevalent clinicopathological condition characterized by excessive accumulation of ectopic lipids within hepatocytes, which in turn leads to significant lipotoxicity. MASLD has become the most common chronic liver disease worldwide, impacting roughly 25% of the global population, and its prevalence is increasing at an alarming rate [1]. MASLD is most often found in people who are obese or have type 2 diabetes, but it can also occur in otherwise healthy individuals without significant risk factors [2, 3]. The development of MASLD is usually associated with high-calorie intake, adipose tissue accumulation, and obesity. With dysregulated lipid metabolism and chronic inflammation serving as its hallmark pathological features, the progression of MASLD may lead to severe metabolic syndrome, including insulin resistance, cirrhosis, and hepatocellular carcinoma. There is no standardized therapeutic agent or effective therapies for MASLD [3, 4], so it is urgent to find new therapeutic targets for MASLD and screen drug candidates based on new targets.

The onset of MASLD is primarily driven by the elevated release of fatty acids from adipose tissue, accompanied by an imbalance between lipid degradation and lipid synthesis, resulting in excessive lipid accumulation. The fatty acid β-oxidation process, happening in the mitochondria and catalyzing the stepwise breakdown of fatty acids, plays a critical role in lipid metabolism. The β-oxidation process involves four sequential enzymatic reactions: dehydrogenation, hydration, oxidation, and thiolysis [5]. The last three reactions of β-oxidation are catalyzed by the mitochondrial trifunctional enzyme, a protein complex composed of two subunits. The trifunctional enzyme α-subunit HADHA carries the 2,3-enoyl-coenzyme A hydratase (ECH) and 3-hydroxyacyl-coenzyme A dehydrogenase (HACD) activities, while the β-subunit HADHB bears the 3-ketoacyl-CoA thiolase (KT) activity.

HADHA has been reported to regulate fatty acid β-oxidation [6], lipid metabolism reprogramming [7], and mitochondrial function [8], as well as being involved in inflammation and oxidative stress [9]. Recent studies have revealed that the acetylation of HADHA regulates hepatic fatty acid oxidation activity and may serve as a key factor in the pathogenesis of fatty liver [10]. HADHA protects against metabolic abnormalities and oxidative stress during the progression of MASLD by inhibiting the activation of the MKK3/MAPK signaling pathway [9]. However, the beneficial effects of HADHA on MASLD have not been adequately studied.

Melatonin (n-acetyl-5-methoxytryptamine) is a multifunctional hormone produced primarily by the pineal gland in response to darkness that was first discovered 60 years ago. Other than the most well-known effect on the regulation of circadian rhythms [11], melatonin has been shown to have a variety of biological effects, including anti-inflammatory [12], antioxidant [13], immune-regulatory [14], and antitumor effects [15]. Recent studies have demonstrated that melatonin plays a significant role in regulating glucose metabolism, enhancing insulin sensitivity [16], modulating lipid metabolism, and promoting weight loss [17]. Additionally, melatonin has been shown to alleviate high-fat diet (HFD)-induced MASLD and reduce oxidative stress [18]. Furthermore, Stacchiotti et al. reported that melatonin effectively mitigates hepatic steatosis in ob/ob mice with MASLD [19]. It has also been reported that melatonin could regulate lipid metabolism in adipocytes and hepatocellular carcinoma cells in vivo and in vitro. In addition, researchers observed that melatonin ameliorated overall metabolic abnormalities in mice chronically fed by HFD [20]. Although the protective effects of melatonin are well known, the direct protein targets and mechanisms of action of melatonin in hepatocytes had not been conclusively identified before.

This study focused on exploring the therapeutic mechanism of melatonin against MASLD and identifying its direct molecular target. Using an HFD-induced mouse MASLD model and palmitic acid-treated mouse hepatocytes, the protective effects of melatonin on hepatic lipid metabolism were verified. Combining mass spectrometry-based proteomics and the cellular thermal shift assay (CETSA) [21–24], HADHA was identified and validated as the direct binding target of melatonin. Mechanistically, melatonin targets HADHA to upregulate peroxisome proliferator-activated receptor gamma coactivator 1-alpha (PGC-1α) expression, promote mitochondrial biogenesis, enhance fatty acid β-oxidation, and regulate the expression of lipid metabolism-related proteins such as acyl coenzyme A oxidase 1 (ACOX1), cluster of differentiation 36 (CD36), and fatty acid synthase (FASN), thereby reducing hepatic lipid accumulation and improving MASLD. Knockdown of HADHA abolished the beneficial effects of melatonin. In conclusion, this study confirms that melatonin ameliorates MASLD by targeting HADHA to regulate mitochondrial function and lipid oxidation, providing a new target and theoretical basis for the treatment of MASLD.

## Results

### Melatonin ameliorates lipid metabolism in HFD-induced MASLD mice

**Fig. 1:**
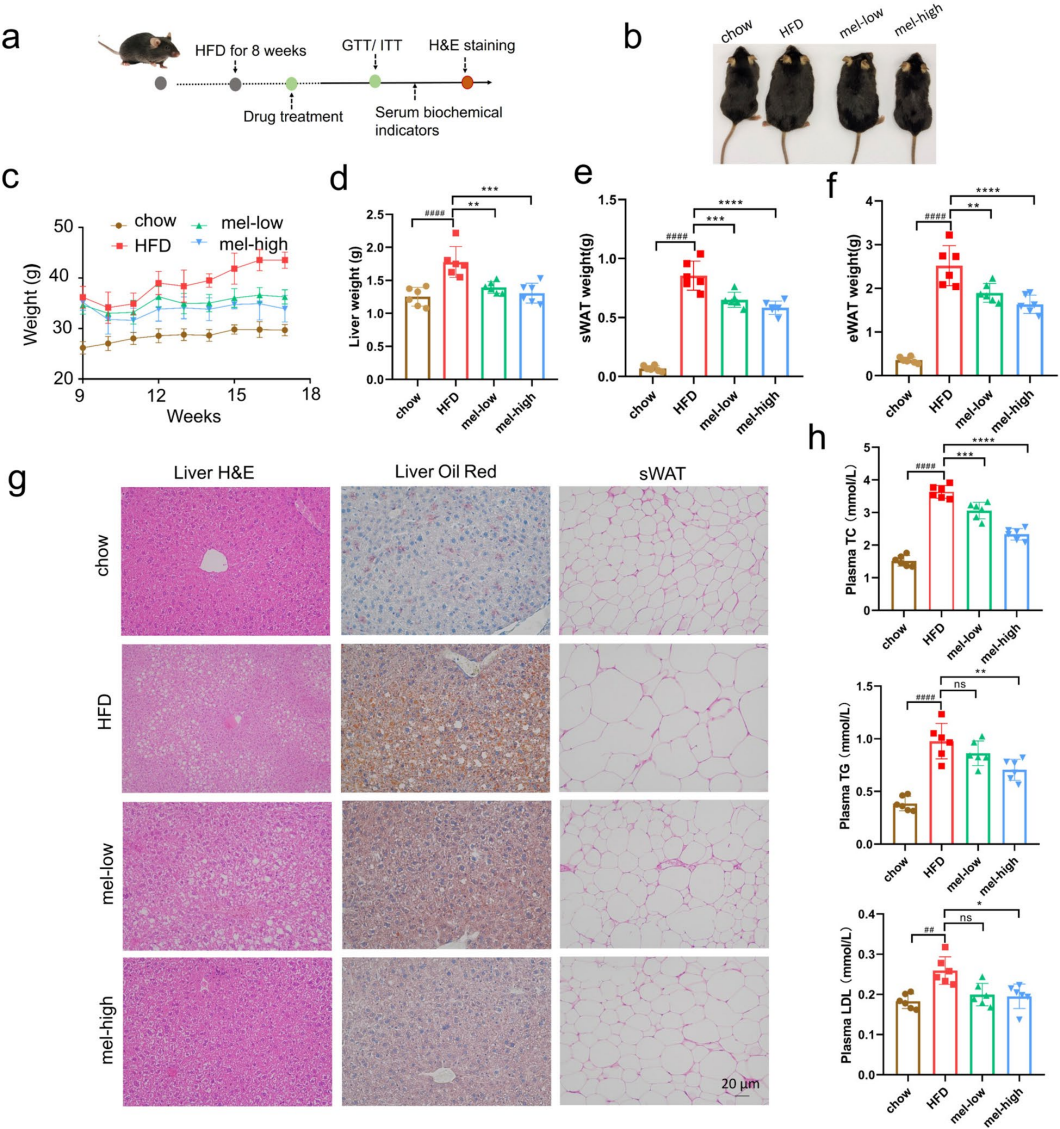
Therapeutic effects of melatonin in metabolic dysfunction-associated steatotic liver disease (MASLD) mice. **a** Experimental design and dosing regimen of mice (*n* = 6); **b** Melatonin significantly reduced the body weight of mice; **c**-**f** Bar plots showing the effects of melatonin on body weight (**c**), liver weight (**d**), subcutaneous fat (sWAT) weight (**e**), and epididymal fat (eWAT) weight (**f**) of HFD mice, *n* = 6; **g** Hematoxylin and eosin (H&E) and oil red staining showing the effects of melatonin on liver and sWAT of mice; **h** Effects of melatonin on the levels of serum total cholesterol (TC), triglyceride (TG), and low-density lipoprotein (LDL) of HFD mice, *n* = 6; chow, control group feeding with a regular chow; HFD, model group feeding with a high fat diet chow; mel-low, melatonin low dose group, 25 mg/kg; mel-high, melatonin high dose group, 150 mg/kg; GTT, glucose tolerance test; ITT, insulin tolerance test. One-way ANOVA followed by Tukey's multiple comparisons test was used for statistical analysis. When compared with the control group, ns: non-significance, #*P* < 0.05, ##*P* < 0.01, ###*P* < 0.001, ####*P* < 0.0001; when compared with the model group, *P < 0.05, **P < 0.01, ***P < 0.001, ****P < 0.0001

We initially aimed to evaluate the pharmacological activities of melatonin against an HFD-induced MASLD mouse model. The scheme for animal experiments is shown in Fig. 1a. Mice were given high-fat chow for eight weeks and induced into a dietary obesity (DIO) model before being randomly divided into a model group (HFD), a melatonin low-dose group (mel-low), and a melatonin high-dose group (mel-high). We found that the body weight and liver weight in the model group were significantly increased compared with the control group fed with a regular chow; whereas the melatonin treatment group showed significantly reduced body weight, liver weight, epididymal fat, and subcutaneous fat weight (Fig. 1b-f).

Furthermore, the model group exhibited significantly higher levels of total cholesterol (TC), serum triglyceride (TG), and low-density lipoprotein (LDL) when compared to the control group (Fig. 1h). In contrast, the melatonin treatment effectively reduced serum levels of TC, TG, and LDL in the model mice (Fig. 1h). Histological analysis with hematoxylin and eosin (H&E) and oil red staining showed that the liver of the control mice had a normal and clear structural morphology and a smooth basement membrane. Conversely, the model group showed the presence of fat vacuoles of varying sizes in the cytoplasm of hepatocytes; the small ones were comparable in size to the tip of a needle, while the large ones squeezed the nucleus to the edge of the cell. This alteration rendered the hepatocytes morphologically similar to adipocytes and led to varying degrees of steatosis, as evidenced by a noticeable yellowish color change in the liver. Melatonin treatment significantly ameliorated these changes. Additionally, melatonin treatment also improved the accumulation of lipid droplets and reduced the size of individual adipocytes in subcutaneous fat (sWAT) in the DIO model (Fig. 1g).

### Effects of melatonin on glucose tolerance, insulin tolerance, and fatty acid metabolism-related protein expression in mice

**Fig. 2:**
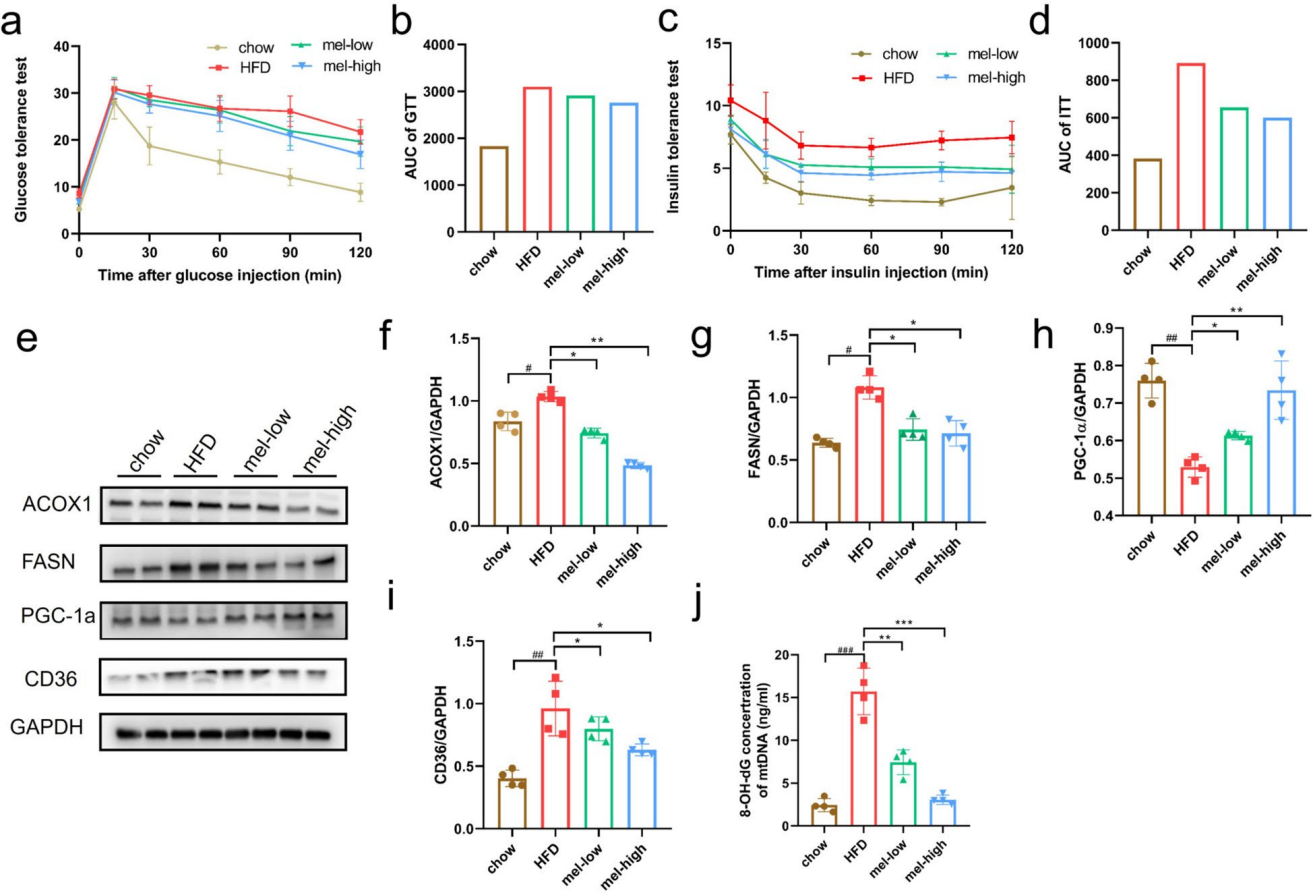
Effects of melatonin on glucose tolerance, insulin tolerance, and fatty acid metabolism-related protein expression in mice (*n* = 6). **a**-**b** Effects of melatonin on glucose tolerance in mice, GTT: glucose tolerance test; **c**-**d** Effects of melatonin on insulin tolerance in mice, ITT: insulin tolerance test; **e**-**i** Animal-level verification of the effects of melatonin on fatty acid metabolism-related protein expression (*n* = 4). **j** Effect of melatonin on oxidative DNA damage of mitochondria. Data are expressed as the mean ± SD, *n* = 4. One-way ANOVA followed by Tukey's multiple comparisons test was used for statistical analysis. Compared with control group, #*P* < 0.05, ##*P* < 0.01, ###*P* < 0.001. Compared with model group, *P < 0.05, **P < 0.01, ***P < 0.001, ****P < 0.0001

In addition, we found that melatonin also decreased fasting blood glucose levels and improved impaired insulin sensitivity and glucose tolerance resulting from hyperlipidemia (Fig. 2a-d). When fatty acid oxidation and lipid export fail to offset the increased hepatic uptake of circulating fatty acids and the hepatic de novo synthesis of fatty acids, MASLD occurs [25]. We then examined several proteins associated with fatty acid synthesis and metabolic pathways. ACOX1, a rate-limiting enzyme of peroxisomal β-oxidation of straight-chain saturated and unsaturated very-long-chain fatty acids, has been reported to increase with fasting or HFD [26]. FASN is a metabolic enzyme that catalyzes fatty acid biosynthesis in cells. Previous studies showed that HFD increases the expression of FASN proteins [27], and decreasing the expression of FASN improves MASLD [28]. Our results revealed that the expression levels of ACOX1 and FASN proteins were substantially elevated in the HFD group and decreased after melatonin administration in vivo (Fig. 2e-g). CD36 is involved in lipid metabolism as a fatty acid transporter protein, and changes in its expression in hepatocytes and adipocytes are a key link in the development of MASLD. Increasing evidence suggests that CD36 also regulates free fatty acid oxidation, lipid synthesis, VLDL secretion, inflammation, and autophagy. The clinical importance of CD36 is further supported by increased levels of CD36 in the livers of MASLD patients [29]. We found that CD36 protein levels were significantly higher in the HFD group than in controls, and that melatonin treatment lowered CD36 expression (Fig. 2e & i). In addition, the levels of PGC-1α protein, a measure of mitochondrial biogenesis, were significantly reduced in the HFD group, and melatonin treatment significantly promoted PGC-1α expression (Fig. 2e & h). To analyze the oxidation products of mtDNA, 8-hydroxydeoxyguanosine (8-OH-dG) was measured in mitochondria. The level of 8-OH-dG was significantly elevated in the cells of the model group compared with the control group, and melatonin treatment dose-dependently decreased the level of 8-OH-dG (Fig. 2j). The above experimental results confirm the successful establishment of the MASLD mouse model and highlight the notable therapeutic effects of melatonin in the mouse model.

### Effects of melatonin on fatty acid metabolism in AML12 cells

**Fig. 3:**
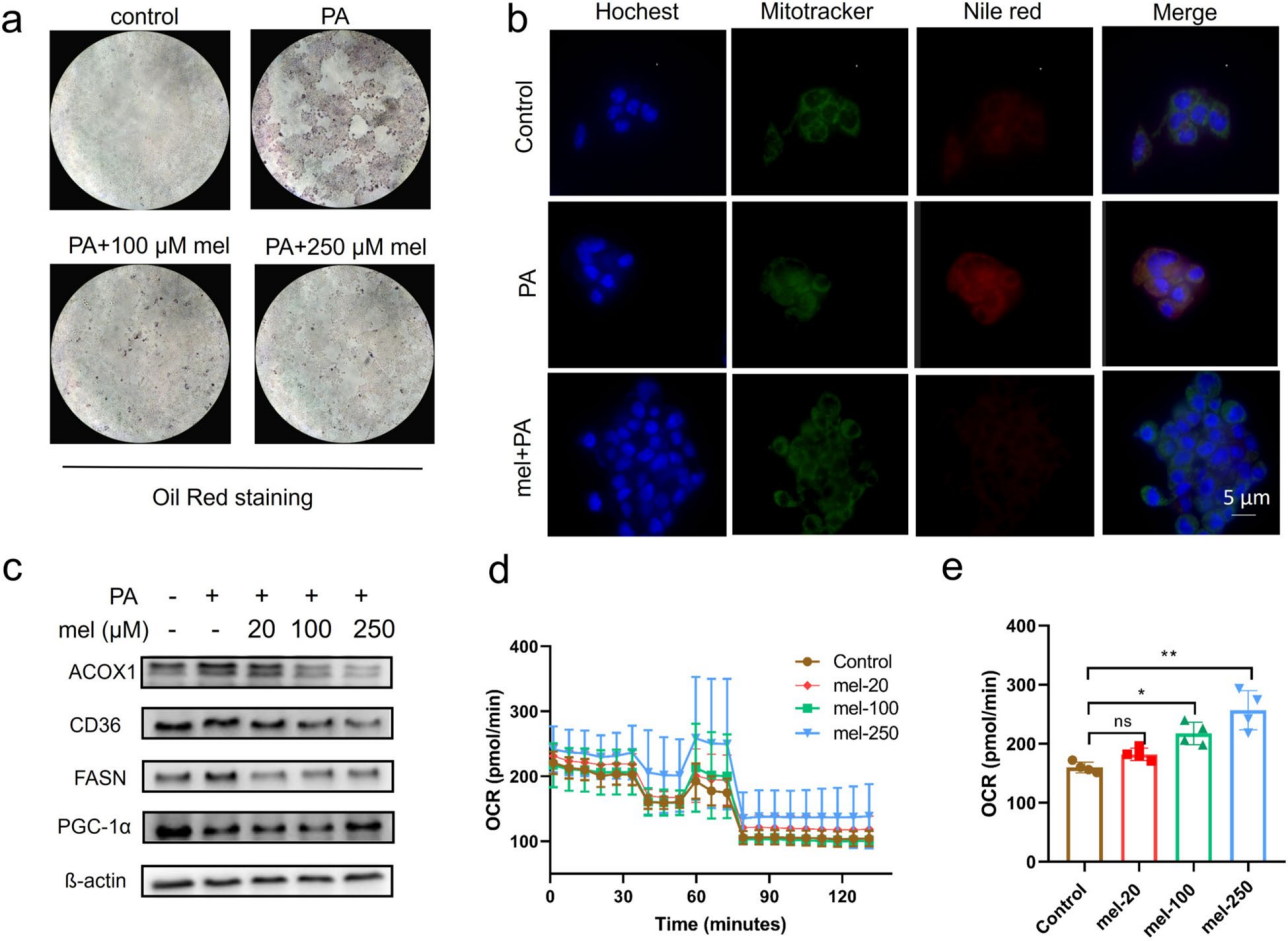
Effects of melatonin on lipid metabolism at the cellular level. **a** Effect of melatonin on lipid droplet formation; **b** Confocal fluorescence showing that melatonin significantly reduced lipid accumulation (Scale bar, 5 µm). **c** Western blots of several proteins related to lipid metabolism, including ACOX1, CD36, FASN, and PGC-1α proteins, in AML12 cells in the presence or absence of melatonin. Data are representative of three independent experiments. **d**-**e** The oxygen consumption rate (OCR) (**d**), maximal respiration (**e**) of AML12 cells treated with or without melatonin, *n* = 4 per group. When compared with the control group, ns: non-significance, *P < 0.05, **P < 0.01, ***P < 0.001

To further investigate the role of melatonin in the treatment of MASLD, we established a palmitic acid (PA)-induced lipid accumulation model in the murine hepatocyte cell line AML12 for in vitro studies. We selected 250 μM as the maximal concentration of melatonin to avoid affecting the viability of AML12 cells. We first examined the effect of different concentrations of melatonin on lipid droplet formation in AML12 cells. Untreated control cells had few lipid droplets, whereas PA-treated cells had massive production of lipid droplets, which were ameliorated by melatonin treatment (Fig. 3a). Similarly, we stained the cells with Nile Red, a selective hydrophobic fluorescent dye for intracellular lipid droplets and neutral lipids, and found that melatonin significantly reduced lipid droplet formation (Fig. 3b). We then examined the effect of melatonin at on the expression levels of proteins related to fatty acid metabolism and synthesis. We found that the resultant changes of protein levels were consistent with the observed changes at the animal level (Fig. 3c). Given that melatonin affects the fatty acid oxidation process, we next examined the effect of melatonin on mitochondrial fatty acid oxidation using the seahorse method, and the results showed that melatonin significantly increased the cellular oxygen consumption rate (OCR) in a concentration-dependent manner, suggesting that melatonin is effective in ameliorating mitochondrial fatty acid oxidation (Fig. 3d-e). The above results further demonstrate that melatonin significantly improves hepatic lipid accumulation and mitochondrial fatty acid oxidation in the PA-induced hepatocyte lipid accumulation model.

### Effects of melatonin on the proteome of liver tissues

**Fig. 4:**
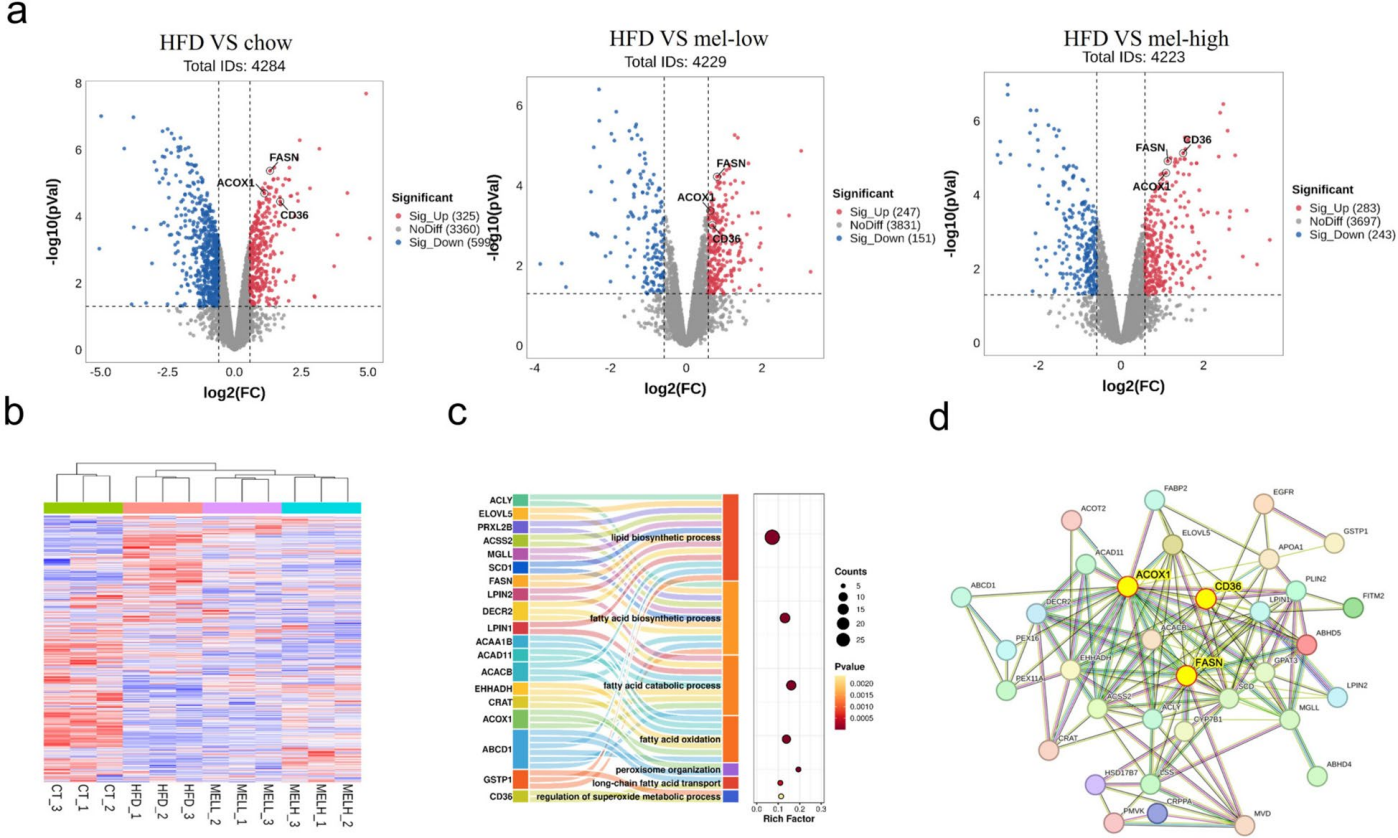
Effect of melatonin on the mouse liver proteome. **a** Volcano plots depicting the differentially expressed proteins (DEPs) in mouse live cells after different treatments, HFD vs chow (left), HFD vs mel-low (middle), and HFD vs mel-high (right), respectively, *n* = 3; **b** Heatmap of total identified proteins.; CT, chow group; HFD, high-fat diet group; MELL, mel-low group; MELH, mel-high group **c** GO pathway enrichment of all the DEPs in HFD vs mel-high; **d** Protein–protein interaction (PPI) analysis of DEPs

Melatonin can regulate lipid metabolism in adipocytes and hepatocellular carcinoma cells in vitro and in vivo [20]. To explore the mechanisms through which melatonin regulates lipid metabolism, we investigated its effects on the mouse liver proteome via proteomics techniques. Our proteomics analysis indicated that the number of differentially expressed proteins (DEPs) increased in a dose-dependent manner after melatonin treatment. There were 924 DEPs (325 up, 599 down) in the model group compared to the control group; 397 DEPs (247 up, 151 down) in the model group compared to the mel-low group, 526 DEPs (283 up, 243 down) in the model group compared to the mel-high group (Fig. 4a). Interestingly, we found that our previously mentioned ACOX1, FASN, and CD36 proteins were among these DEPs. The heatmap of total identified proteins is shown in Fig. 4b. Gene Ontology (GO) enrichment analysis of DEPs were performed, and the results suggested that a total of 526 DEPs were mainly involved in lipid biosynthetic process, fatty acid biosynthetic process, inflammatory response, fatty acid catabolic process, fatty acid oxidation, and long-chain fatty acid transport processes, further suggesting that melatonin can regulate fatty acid metabolic processes (Fig. 4c). We also performed protein–protein interaction (PPI) network analysis of DEPs involved in these pathways, and the results showed that several key proteins involved in fatty acid metabolism such as ACOX1, FASN, and CD36 possess highly complex and direct interactions (Fig. 4d). In summary, these results revealed that melatonin affects the expression of proteins related to fatty acid metabolism.

### Identification of melatonin targets using CETSA-MS

**Fig. 5:**
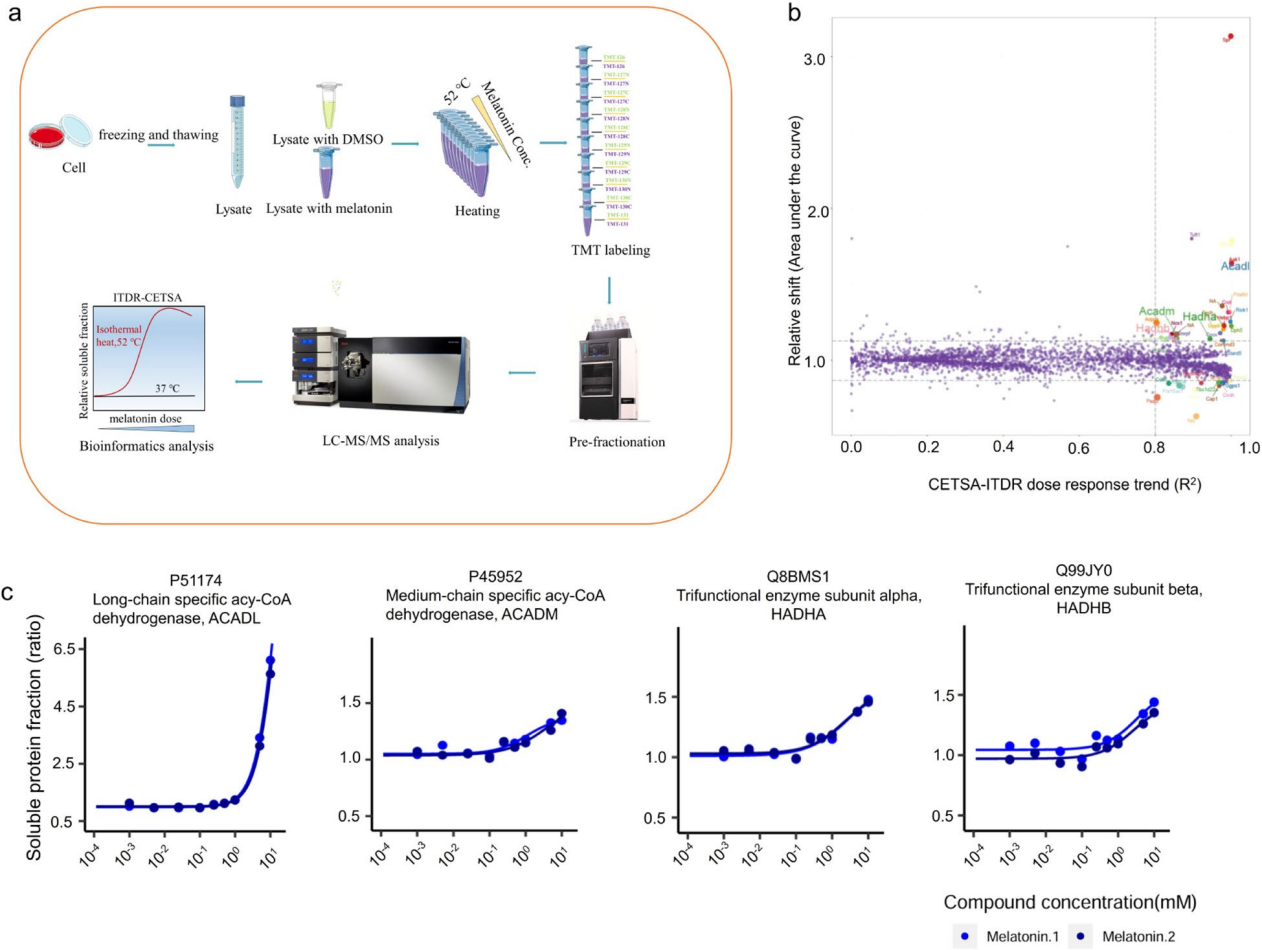
Cellular thermal shift assay coupled to mass spectrometry (CETSA-MS) to identify direct binding proteins of melatonin in 3T3-L1 cells. **a** Flowchart of the CETSA-MS experiment used for target identification; **b** An R^2^ (R-squared) area under the curve (R.^2^-AUC) plot showing the changes in thermal stability of the whole proteome proteins under different melatonin concentrations; **c** Isothermal dose–response (ITDR) curve-based thermal shift profiles of four potential target proteins (*n* = 2)

Cellular thermal shift assay coupled to mass spectrometry (CETSA-MS) is a label-free method that can directly detect the binding of drugs or compounds to target proteins at the proteome level [22, 23]. By monitoring the effect of different concentrations (0–10 mmol/L) of melatonin on the thermal stability of the proteome under the 52 °C heating conditions (Fig. 5a), we identified 32 candidate interacting proteins from a total of 4560 quantified proteins (Fig. 5b). Among these, proteins related to lipid metabolism and β-fatty acid oxidation included long-chain specific acyl-CoA dehydrogenase (ACADL), medium-chain specific acyl-CoA dehydrogenase (ACADM), and the mitochondrial trifunctional enzyme subunit alpha and beta (HADHA and HADHB) (Fig. 5b and c). These enzymes play crucial roles in lipid metabolism and fatty acid oxidation. These results revealed that melatonin may ameliorate MASLD by targeting these key proteins, thereby affecting fatty acid metabolism.

### Validation of target proteins and the effect of melatonin on target protein expression

**Fig. 6:**
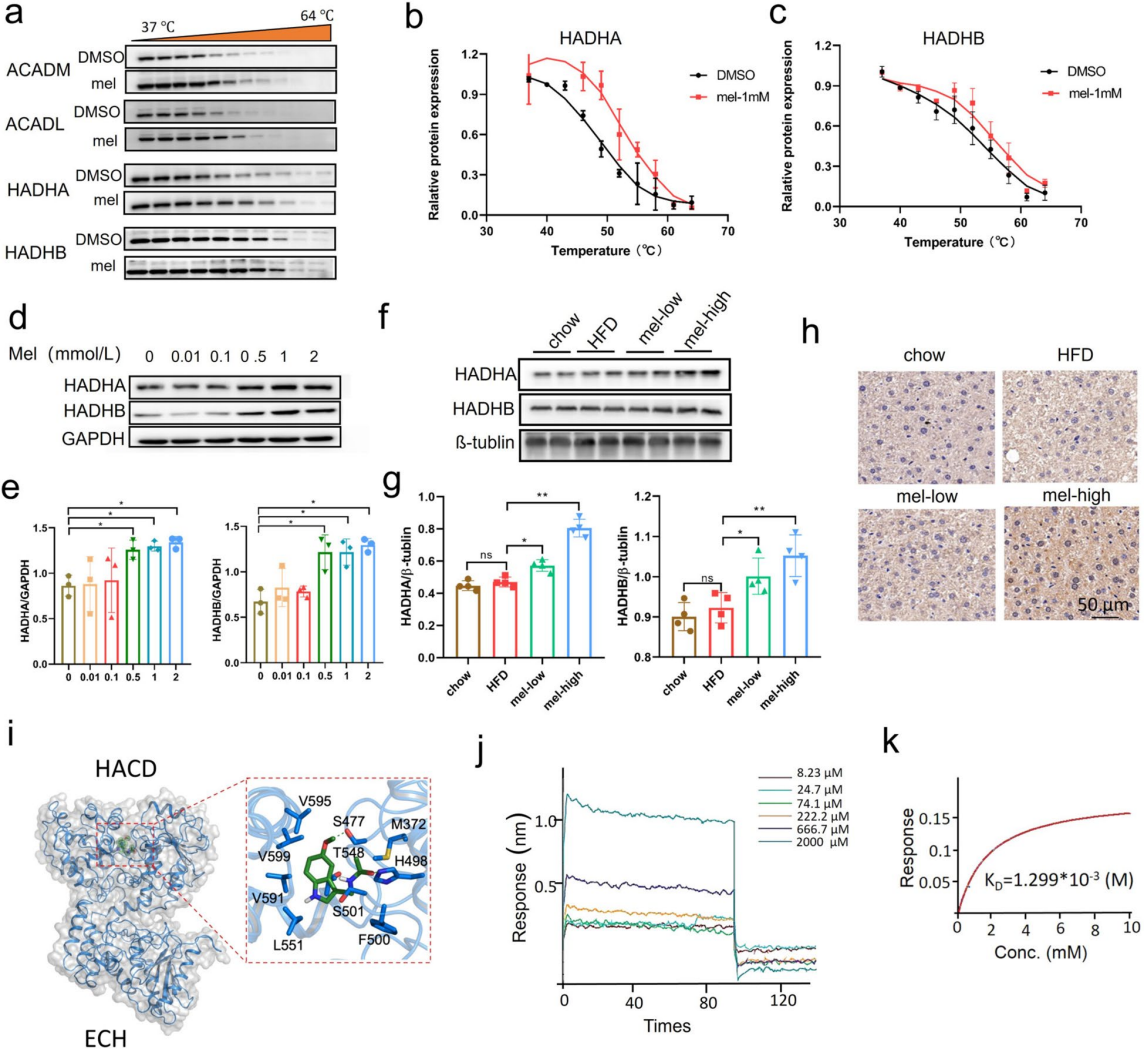
Cellular thermal shift assay coupled to western blotting (CETSA-WB) analysis verifying the direct interaction between melatonin and HADHA protein (*n* = 3). **a**-**c** CETSA-WB analysis verifying the direct interaction between melatonin and HADHA or HADHB (*n* = 3). **d**-**e** Western blot method to detect the expression of HADHA and HADHB proteins under different melatonin concentration conditions (*n* = 3). **f**-**g** Western blot analysis of changes in HADHA and HADHB protein levels in liver tissues after melatonin treatment at different doses (*n* = 4). **h** Immunohistochemical staining of mouse liver tissues with a monoclonal antibody against HADHA (scale bar, 50 µm). **i** Molecular docking of melatonin with HADHA protein. **j**-**k** Bio-layer interferometry (BLI)-based measurement of the affinity between melatonin and HADHA. One-way ANOVA followed by Tukey's multiple comparisons test was used for statistical analysis. ns: Non-significance, #*P* < 0.05, ##*P* < 0.01, ###*P* < 0.001; *P < 0.05, **P < 0.01, ***P < 0.001

Next, we performed the cellular thermal shift assay coupled to Western blotting (CETSA-WB) experiments in cell lysate to validate the direct interactions of melatonin with the candidate target proteins (Fig. 6a). Protein extracts from 3T3-L1 cells were incubated with melatonin (1 mmol/L) or DMSO and heat-pulsed over a range of 37 °C to 64 °C, then soluble proteins were extracted and the target proteins were quantified by Western blot. Interestingly, ACADL, ACADM, HADHA, and HADHB all showed significant thermal stability in the melatonin-treated group (Fig. 6a-c). The above findings suggest that melatonin may directly bind to ACADL, ACADM, HADHA, and HADHB. Studies indicated that HADHA is closely associated with MASLD [9, 10], so we focused our subsequent research on the HADHA protein.

Furthermore, we examined the effects of melatonin on the expression levels of HADHA and HADHB proteins at both the cellular and animal levels. It was found that melatonin significantly increased the expression of HADHA and HADHB (Fig. 6d-g). Immunohistochemistry further confirmed the effect of melatonin on upregulating the HADHA protein expression in liver tissue (Fig. 6h).

We then performed a molecular docking analysis between melatonin and the HADHA protein. The docking results indicate that melatonin interacts with HADHA at the HACD regions. In the active sites of HACD (Fig. 6i), melatonin is surrounded by a hydrophobic cluster composed of M372, F500, L551, V591, V599, and V595. Several hydrophilic residues, including T548 and S501, are located near the ligand. Two polar bonds are formed between S477 and H498 with melatonin (Fig. 6i). Next, we examined the affinity of HADHA proteins for melatonin by bio-layer interferometry (BLI). Results showed that the proteins bound and dissociated from melatonin with a *K*_D_ value of 1.299 mM (Fig. 6j-k). These results suggested that melatonin may improve β-fatty acid oxidation by targeting HADHA, simultaneously increasing its expression, thereby enhancing lipid oxidation and improving the treatment of MASLD.

### Knockdown of HADHA improves lipid metabolism

**Fig. 7:**
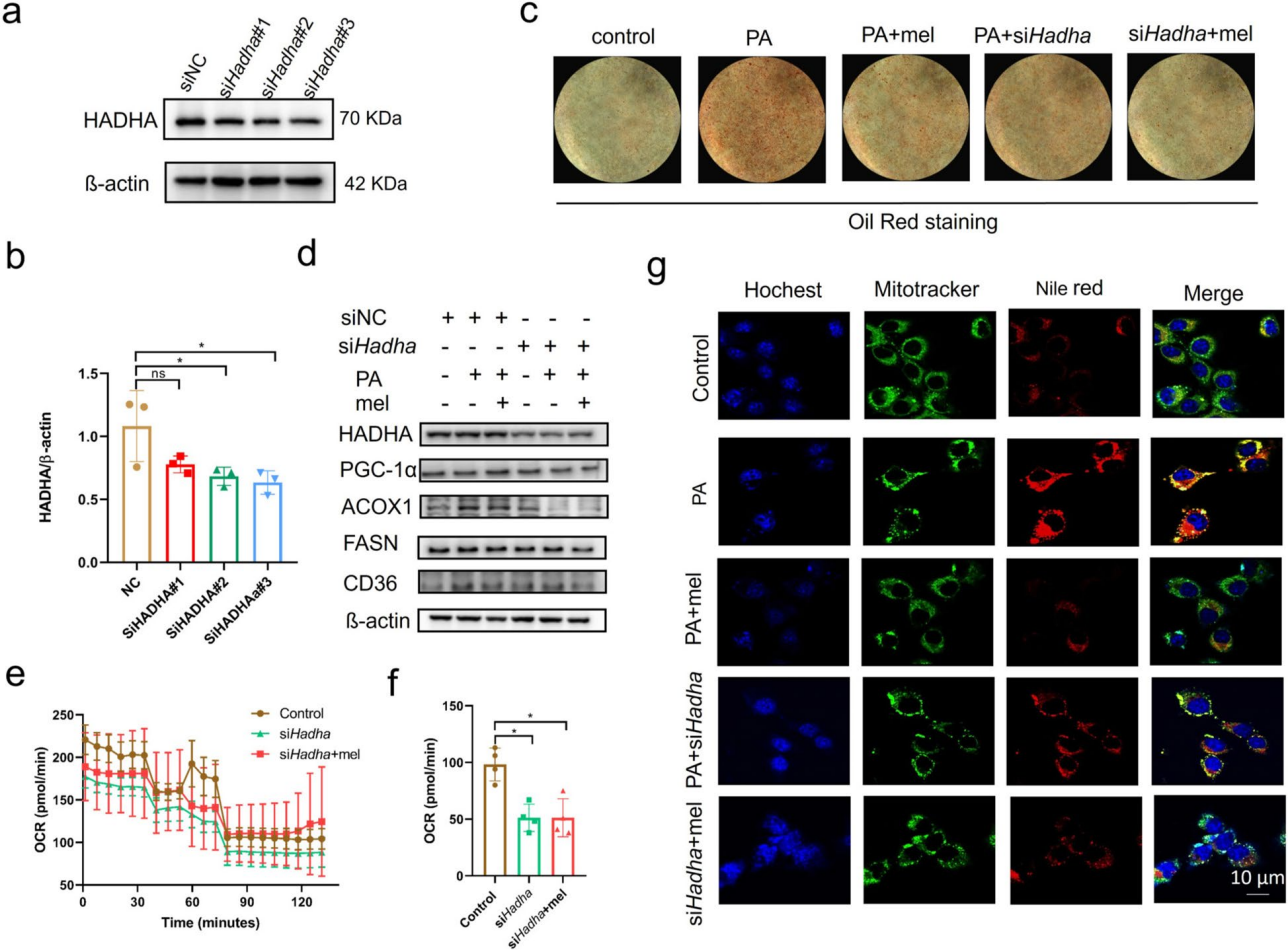
The effect of melatonin after knockdown of HADHA. **a**-**b** Western blots levels of HADHA in AML12 cells transfected with si*Hadha*#1, si*Hadha*#2, or si*Hadha* #3 (*n* = 3). **c** Lipid droplet formation experiments of AML12 cells transfected with si*Hadha*#3. **d** Western blot analysis of changes in the levels of proteins associated with fatty acid signaling pathways in AML12 cells after transfection with si*Hadha*#3. **e**–**f** The oxygen consumption rate (OCR) (**e**) and maximal respiration (**f**) of 3T3-L1 cells transfected with si*Hadha*#3. *n* = 3 per group. **g** Confocal fluorescence experiments of AML12 cells transfected with si*Hadha*#3. One-way ANOVA followed by Tukey's multiple comparisons test was used for statistical analysis. ns: Non-significance, *P < 0.05, **P < 0.01, ***P < 0.001

To verify the role of HADHA (encoded by the *Hadha* gene in mouse) in lipid accumulation and metabolism, we transfected three *Hadha*-targeting siRNAs (si*Hadha* #1, si*Hadha* #2, and si*Hadha* #3) into AML12 cells, and all three siRNAs resulted in a significant decrease in HADHA expression (Fig. 7a-b). As expected, oil-red and confocal microscopy experiments showed a reduction in lipid accumulation after knockdown of HADHA. The improvement in lipid accumulation was not significant after melatonin administration. The results from both confocal binding and oil-red staining were consistent, suggesting that melatonin’s improvement of lipid metabolism in MASLD is mediated by HADHA (Fig. 7c & g). Furthermore, enzymes related to lipid synthesis and metabolism, including FASN, CD36 and ACOX1 proteins, were not elevated by PA induction after knockdown of HADHA, and the effect on the expression of these proteins after administration was not significant affected (Fig. 7d). Additionally, knockdown of HADHA decreased the level of OCR, and the OCR values were not significantly elevated after the administration of melatonin, confirming the role of HADHA in mitochondrial fatty acid metabolism (Fig. 7e & f). In conclusion, these results suggest that knockdown of the HADHA protein significantly inhibits lipid oxidation and accumulation, thereby attenuating PA-induced lipid droplet formation, while melatonin exerts its therapeutic effects on MASLD through HADHA-mediated lipid synthesis and metabolism.

## Discussion

MASLD is a growing global health issue, increasingly linked to cirrhosis and hepatocellular carcinoma [30]. While several molecular pathways involved in its progression have been identified, the exact initial cause of MASLD remains unclear, and effective treatments are still limited [31]. However, studies have shown that melatonin, an endogenous hormone in mammals, can prevent MASLD, protect liver function in individuals with obesity and diabetes, and slow its progression by reducing hepatic steatosis, inflammation, and fibrosis [32]. These benefits may be due to melatonin’s effects on oxidative stress, mitochondrial function, and inflammation [33–35], though its mechanisms likely extend beyond these pathways. Previous studies have demonstrated that melatonin inhibits cell division by blocking the NR4A1/DNA-PKcs/p53 pathway while restoring mitochondrial autophagy, ultimately improving mitochondrial and hepatic function in MASLD [34]. Furthermore, studies indicated that melatonin increases PGC-1α protein expression, thereby enhancing mitochondrial oxidative phosphorylation capacity in a PGC-1α-dependent manner [36].

Our study revealed that melatonin significantly reduces serum cholesterol, triglyceride, body weight, and adiposity in HFD-induced mice. Numerous investigations on MASLD have indicated that disorders in hepatocyte metabolism are the main contributors to the disease [33]. Our proteomics analysis revealed that DEPs are highly enriched in lipid biosynthesis, fatty acid metabolism, and inflammatory responses through GO enrichment analysis. Additionally, DEPs were also distinctly associated with mitochondrial and peroxisomal functions. These findings suggest that melatonin preferentially targets fatty acid metabolism, which is critically governed by mitochondria and peroxisomes—organelles central to metabolic catalysis. Moreover, our results demonstrate that melatonin enhances fatty acid oxidation and lipid metabolism while reducing hepatic lipid accumulation in both cellular and murine models of MASLD. Taken together, these findings underscore the therapeutic potential of melatonin in a mouse model of MASLD by promoting hepatic lipid metabolism.

Mitochondrial fatty acid β-oxidation is the main pathway of fatty acid catabolism. Increasing fatty acid β-oxidation and decreasing lipid accumulation may be a therapeutic strategy for MASLD [34]. To determine the mechanism of lipid accumulation suppressed by melatonin, we evaluated mitochondrial respiratory capacity using a Seahorse SF analyzer. The OCR measurements demonstrated that melatonin treatment dose-dependently enhanced mitochondrial respiration in treated cells compared to the control cells, indicating its role in stimulating oxidative metabolism. Using the CETSA-MS methodology, we identified HADHA, HADHB, ACADL, and ACADM, which are key enzymes in mitochondrial and peroxisomal fatty acid β-oxidation, as direct binding proteins of melatonin. The four proteins HADHA, HADHB, ACADL, and ACADM, as core executors of fatty acid β-oxidation, play a pivotal role in the progression of MASLD. Given the critical importance of the HADHA protein in MASLD, our work primarily focuses on the relationship between melatonin and the HADHA protein. These interactions were validated in vitro via CETSA-WB and corroborated in vivo through expression profiling. Mechanistically, melatonin significantly increased the expression levels of HADHA and HADHB while suppressing the expression of ACOX1, FASN, and CD36. Simultaneously, melatonin upregulates PGC-1α, which drives mitochondrial biogenesis and β-oxidation. This dual regulatory effect underpins melatonin’s therapeutic efficacy in restoring metabolic homeostasis in MASLD. HADHA knockdown abrogated melatonin-induced increases in mitochondrial OCR and the expression of related proteins, demonstrating that melatonin suppresses lipid accumulation and drives mitochondrial biogenesis through direct interaction with HADHA and other target proteins.

It is well known that melatonin itself is a potent free radical scavenger and broad-spectrum antioxidant capable of directly neutralizing reactive oxygen and nitrogen species [13]. Simultaneously, it exhibits significant anti-inflammatory properties, alleviating neuroinflammation by inhibiting NLRP3 inflammasome and downregulating NF-κB pathways [12]. These distinct antioxidant and anti-inflammatory pathways, alongside the mechanism revealed in this study—enhancing fatty acid oxidation and improving mitochondrial function by targeting HADHA expression— may operate in parallel and interact synergistically. Together, they form a comprehensive picture of melatonin’s multi-layered, multi-targeted mode of action.

Despite the novel insights into melatonin’s direct targeting of HADHA and its regulatory effects on mitochondrial fatty acid β‑oxidation in MASLD, this study has several limitations. First, all mechanistic experiments were performed exclusively in cellular models and HFD‑induced mice, which cannot fully recapitulate the complex genetic, metabolic, and environmental heterogeneity of human MASLD. Second, the optimal dosage, duration, and long‑term safety profile of melatonin for treating MASLD remain undetermined, as only a limited range of concentrations and treatment periods were evaluated. Furthermore, although melatonin has demonstrated significant effects in the mouse MASLD model, our study lacked human tissue and clinical samples.

**Fig. 8:**
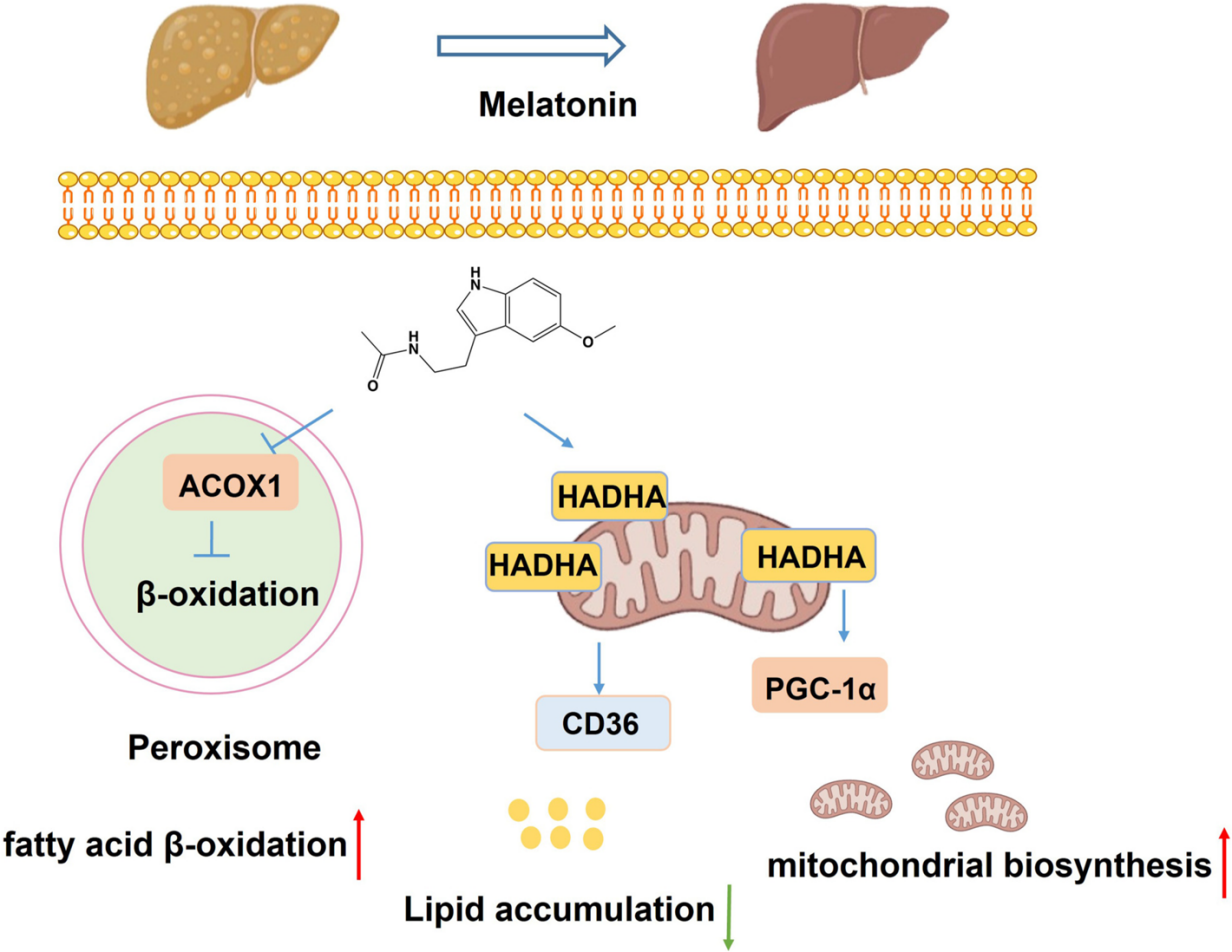
Schematic description of melatonin targets mitochondrial trifunctional enzyme HADHA to improve lipid metabolism in metabolic dysfunction-associated steatotic liver disease (MASLD)

In conclusion, by integrating quantitative proteomics and CETSA methodology, we identified and validated HADHA as a direct molecular target of melatonin. It exerts its regulatory role in lipid metabolism by modulating the expression of other lipid metabolism-related proteins, elucidating the mechanism by which it restores metabolic homeostasis (Fig. 8). Our findings provide a theoretical and mechanistic foundation for melatonin research in MASLD and offer a framework for future studies targeting mitochondrial lipid oxidation pathways.

## Materials and methods

### Cell culture and viability assay

3T3-L1 and AML12 cells were obtained from the Chinese Academy of Medical Sciences (Beijing, China). Cells were cultured in Dulbecco's modified Eagle's medium (Gibco, New York, USA) with 10% fetal bovine serum (Gibco, New York, USA) and incubated at 37 °C with a 5% CO_2_ humidified atmosphere. 3T3-L1 cells were differentiated and subsequently treated with melatonin for 24 h, after which the expression levels of target proteins were analyzed. Cells were inoculated into 96-well plates at a density of 6 × 10^3^ cells/well overnight and then incubated with different concentrations of melatonin for 24 h. Cell viability was assessed using the CCK-8 kit following the manufacturer’s protocol.

### Animal experiments

All animal experimental procedures were approved by the Chinese Committee for the Care and Use of Animals and the Committee for the Care and Use of Laboratory Animals of the Chinese Academy of Traditional Chinese Medicine (Beijing, China). After one week of acclimatization, C57BL/6 J (male, *n* = 6, 22 ± 3 g; Weitong Lihua Laboratory Animal Technology Co, Beijing, China) mice were randomly divided into a control group and a model group. The control group was given normal chow for 8 weeks, and the model group was given 60% high-fat chow for 8 weeks. The model mice were then randomly divided into three groups: the model group, the melatonin low-dose group (25 mg/kg), and the melatonin high-dose group (150 mg/kg). The drug was administered by gavage once a day for 8 weeks. Blood collection and dissection were performed on mice anesthetized with sodium pentobarbital.

One week before dissection, the mice were subjected to an insulin tolerance test (ITT) and a glucose tolerance test (GTT). For the glucose tolerance test, mice were fasted for 12 h before receiving an intraperitoneal injection of glucose and monitoring blood glucose levels using a portable glucose monitor (Yuyue, Jiangsu, China). Blood samples were collected from the tail tip at 0, 15, 30, 60, 90, and 120 min post-glucose injection to measure and record blood glucose concentrations. Similarly, in the insulin tolerance test, mice fasted overnight for 6 h before receiving an intraperitoneal injection of insulin, with blood glucose levels monitored as described above. Livers were collected for subsequent proteomics experiments.

### Biochemical and histological assay

Serum total cholesterol (TC), triglyceride (TG), and low-density lipoprotein (LDL) levels were analysed using commercial kits (A111-1–1; A110-1–1 and A113-1–1, respectively, Jiancheng Bioengineering Institute, Nanjing, China) according to the manufacturer’s instructions and detected using a biochemical analyzer (MaiRui, BS420, China). Liver tissue samples from mice were embedded in paraffin, sectioned, and subjected to hematoxylin and eosin (H&E) staining as well as oil red O staining to assess histological changes. Subcutaneous and epididymal fat were cryosectioned using frozen sections and subjected to H&E staining for the effect of drugs on fat size and morphology.

### Identification of the target proteins of melatonin by CETSA-MS

CETSA (Cellular Thermal Shift Assay) is an experimental method that leverages the principle that “ligand binding enhances the thermal stability of target protein”. It enables the direct assessment of ligand-target interactions under near-physiological conditions, effectively addressing the limitation of traditional in vitro binding assays, which struggle to reflect true in vivo binding scenarios [22]. The target proteins of melatonin were identified using CETSA-MS, which was carried out following established protocols in the laboratory [37, 38]. Differentiated 3T3 cells were harvested. The cell pellet was lysed using a mild lysis buffer, which contains 50 mmol/L HEPES, 0.1 mmol/L activated Na₃VO₄, 5 mmol/L β-glycerophosphate, 1 mmol/L Tris(2-carboxyethyl)phosphine (TCEP), 20 mmol/L MgCl₂ and 1 × protease inhibitor (Thermo, USA), and a mild lysis method (three freeze–thaw cycles between liquid nitrogen and room-temperature water) was employed to preserve the activity of most proteins.

Then the resulting cell lysates were treated with different concentrations of melatonin (9 concentration gradients of 0.001, 0.025, 0.01, 0.1, 0.5, 1, 2.5, 5, and 10 mM) as well as a DMSO control to serve as a negative control. The cell lysates underwent thermal cycling at 52℃ for 3 min each, followed by a cooling period at 4℃ for an additional 3 min on a PCR instrument. The lysates were then centrifuged at 4℃ for 10 min at 20,000 g. The supernatants subsequently underwent reduction with dithiothreitol (DTT, 20 mM), alkylation with iodoacetamide (IAA, 50 mM), and overnight trypsin digestion (the ratio of trypsin to protein is 1:100). The peptide samples were reconstituted and labeled with TMT10plex reagent, then the samples were desalted using an Oasis HLB column and dried. To enhance the analytical separation, the peptides were subjected to high-performance liquid chromatography (HPLC)-based fractionation, spun-dried, and then reconstituted in a solution containing 0.1% formic acid and 1% acetonitrile. These 20 fractionations were analyzed by a high-resolution Orbitrap LC–MS/MS mass spectrometer, with resultant data processed through the Proteome Discoverer software. LC–MS/MS instrument parameters and mass spectrometry settings were the same as previously reported [38] and with the details provided in the Supplementary Materials. Based on the protein quantification results, isothermal dose–response (ITDR) curves were plotted using the mineCETSA package [39].

### Liver tissue mass spectrometry sample preparation and proteomic analysis

Liver tissue samples (*n* = 3) from the chow, HFD, mel-low, and mel-high were separately added to the RIPA solution and protease inhibitor. Tissue homogenizers were used for homogenization. After thorough mixing, samples were centrifuged at 4 °C at 22,000 g for 15 min. The supernatant was collected, and protein concentration was measured. From each sample, 100 µg of protein was taken and mixed with a final concentration of 40 mM DTT. After treatment at 37 °C for 30 min, a final concentration of 50 mM IAA was added and incubated in the dark for 30 min. The mixture was precipitated with acetone at −40 °C for 30 min, centrifuged at 22,000 g for 15 min. The pellets were solubilized with 100 mM TEAB with the assistance of sonication, then digested with trypsin for 16 h. The samples were then passed through a desalting column (WAT094225, Waters, USA) and dried. Then reconstituted in a solution containing 0.1% formic acid and 1% acetonitrile. These samples were analyzed by a high-resolution Orbitrap LC–MS/MS mass spectrometer.

The processing of raw data followed our previously published article with minor modifications [40]. Detailed methods are provided in the Supplementary Materials. The mass spectrometry proteomics data have been deposited to the ProteomeXchange Consortium via the PRIDE partner repository with the dataset identifier PXD071550 [41].

### Western bolting

Total proteins from 3T3-L1 cells were extracted by RIPA lysis buffer containing protease inhibitors, separated by SDS-PAGE, and electrotransferred onto polyvinylidene difluoride (PVDF) membranes. The membranes were incubated with primary antibodies, including ACOX1 (10,957–1-AP, Proteintech, China), FASN (2,001,194, Zen-bioscience, China), CD36 (18,836–1-AP, Proteintech, China), PGC-1α (2178S, cell signaling, USA), HADHA (ab203114, Abcam, UK), HADHB (ab230667, Abcam, UK), ACADL (ab128566, Abcam, UK), ACADM (ab108192, Abcam, UK), GAPDH (R380626, Zen-bioscience, China), Tubulin (M1501-1, HuaBio, China) and β-Actin (AC026, ABclonal, China) overnight at 4 °C, after 3 times TBST washing, the membranes were incubated with the corresponding secondary antibody (Anti-rabbit IgG, HRP-linked Antibody, 7074 s, cell signaling, USA; Anti-mouse IgG, HRP-linked Antibody, 7076 s, cell signaling, USA). All primary antibodies were diluted at a ratio of 1:1000, while secondary antibodies were diluted at a ratio of 1:3000. Protein bands were observed and analyzed using ImageJ software.

For CETSA-WB, experiments were conducted following a previously established protocol, with minor modifications [37]. 3T3-L1 cells were collected in 100-mm dishes with PBS containing protease inhibitors. Cells were subjected to freezing and thawing cycles in liquid nitrogen and repeatedly mechanically pulverized, and cell lysate supernatants were obtained by centrifugation. An equal amount of supernatant protein (1 mg) was then treated with DMSO or melatonin (1 mM) for 1 h and gently shaken at a constant temperature. The treated supernatant was divided into 10 aliquots and heated at the indicated temperatures. The cooled samples were centrifuged again to obtain the supernatant and analyzed by Western blotting (WB).

### RNA interference and transfection

Three siRNA sequences targeting the mouse *Hadha* gene were designed and synthesized by Ruibo Xingke (Beijing, China). The siRNA sequences and negative control sequences are listed in Table S1. These siRNAs or negative control (NC) were transfected into AML12 cells using Lipofectamine 3000.

### Seahorse XF-96 metabolic flux analysis

Cells were inoculated on an XF-96 plate at a density of 3 × 10^4^ cells/well. The medium was replaced with 600 μL substrate-limited medium at 6 h before metabolic flux analysis, and then replaced with 410 μL FAO Assay medium before re-analysis and cultured in a CO_2_-free incubator at 37℃ for 45 min. During the experiment, palmitate-BSA (175 μM), BSA (175 μM), etomoxil (80 μM), antimycin A (0.2 μM), and rotenone (0.2 μM) inhibitors were injected successively. The oxygen consumption rate (OCR) is then calculated automatically by the Seahorse XF-96 analyzer (Seahorse Bioscience, CA, USA).

### Docking analysis

Molecular docking is a computational technique that identifies the most stable and likely complex structures by sampling possible binding orientations (conformations) and scoring them based on shape complementarity and interaction energies. The 3D structure of the human mitochondrial trifunctional protein (TFP) complex was downloaded from the Protein Data Bank (PDB ID: 5ZQZ). One TFPα subunit/HADHA was isolated for molecular docking analysis. Before docking, missing loop structures were added to the model using a predicted structure from the AlphaFold2 database. Using Vina in the DockingPie tool (version 1.2) integrated with PyMOL (version 3.1), hydrogen atoms were added to the receptor before analysis. The 3D structure of melatonin is generated and optimized using the Avogadro platform. After preparing the ligand and receptor, the docking grid and parameters were set according to the active sites of both ECH and HACD domains. The top-scoring conformations from the docking poses were selected for clustering, and the binding poses from the optimal conformations were presented for the binding sites.

### Statistical analysis

Statistical analyses were performed using one-way ANOVA in GraphPad Prism 8 unless otherwise stated, and differences between two groups were assessed for significance by Student's t-test. Statistical significance was defined as a P value of less than 0.05. All data are expressed as mean ± standard error of the mean (SEM) of at least 3 biological replicates.

## Supplementary Information

Supplementary Material 1.

## Data Availability

The mass spectrometry proteomics data have been deposited to the ProteomeXchange Consortium via the PRIDE partner repository with the dataset identifier PXD071550.
