# Supplementary material for: Melatonin targets mitochondrial trifunctional enzyme HADHA to improve lipid metabolism in metabolic dysfunction-associated steatotic liver disease
Source: Mol Biomed. 2026 May 12;7:68. doi: 10.1186/s43556-026-00461-0 (PMC13168447; doi:10.1186/s43556-026-00461-0)
Supplement: Supplementary file 1 — Supplementary Material 1. [file 43556_2026_461_MOESM1_ESM.docx]

**Melatonin targets mitochondrial trifunctional enzyme HADHA to improve lipid metabolism in metabolic dysfunction-associated steatotic liver disease**

Yongping Zhu^1#^, Yanqing Liu^1#^, Rui Liu^2#^, Junzhe Zhang^1#^, Yuqing Meng^1^, Li Liu^1^, Xin Liu^3^, Dandan Liu^1^, Liwei Gu^1^, Linying Zhong^1^, Xianyu Xu^4^, Ying Li^5^, Jinyan Xu^6^, Lingyun Dai^3*^, Shengnan Shen^1*^, Jigang Wang^1,4,7*^

**Supplementary Materials**

**LC-MS/MS analysis**

Peptide samples were injected into the Ultimate 3000 RSLC nano system (Thermo Fisher Scientific), which was coupled with the Orbitrap Fusion Lumos Tribrid mass spectrometer (equipped with FAIMS Pro interface, Thermo Fisher Scientific) for analysis. Peptides were loaded onto a trapping column (Acclaim PepMap 100 C18, 75 μm × 20 mm, 3 μm, Thermo Scientific,164535) at a flow rate of 5 μL/min using the loading solvent (aqueous solution containing 0.1% formic acid and 2% acetonitrile) for 4 minutes. Subsequently, the samples was separated on the analytical column (Acclaim PepMap RSLC C18, 75 μm × 250 mm, 2 μm, Thermo Scientific,164941) at 300 nL/min using the following gradient: 2-4% B (Buffer B) from 0-4 min, 4-8% from 4-8 min, 8 % to 35% between 8–58 min, B buffer concentration increased from 35% to 90% between 58–62 min, B buffer concentration maintained at 90% between 62–67 min (Buffer A: 0.1% formaldehyde (FA); Buffer B: 0.1% FA, 80% acetonitrile). The FAIMS Pro interface operated with default parameters, switching the compensation voltage (CV) between -40V, -60V, and -80V, with a cycle time of 3 seconds per CV. Spectra were acquired in Orbitrap mode at a resolution of 50,000, with a mass range of 100–500 m/z, a standardized AGC target value of 250%, and a maximum injection time of 100 milliseconds.

**Proteomic data analysis**

Data were processed using Proteome Discovery software (Thermo Fisher Scientific, version 2.4), Mascot (v2.6.1), and the SEQUEST® HT search engine. All mass spectrometry data were searched against the UniProtKB mouse FASTA database (retrieved on 2022 May 15). Identification search parameters were set as follows: MS1 tolerance 10 ppm; MS/MS tolerance 0.02 Da; trypsin as the specific digestion enzyme with 2 maximum missed sites allowed. Carbamidomethylation (C) was set as static modification, and oxidation (M), met-loss (M) and acetyl (N-terminus) were set as variable modifications. For protein identification, the false discovery rate (FDR) for PSMs was set to 1%. Following protein identification, the proteomics dataset was normalized by total peptides amounts and visualized using statistical software R (version 4.1.1). Differentially Expressed Proteins (DEPs) Identification: The limma package (version 3.54.2) was used to calculate P-values for expression difference and fold change values. Proteins with P < 0.05 and Fold Change > 1.5 were selected as DEPs. Enrichment analysis of DEPs was performed using GO and KEGG resources.


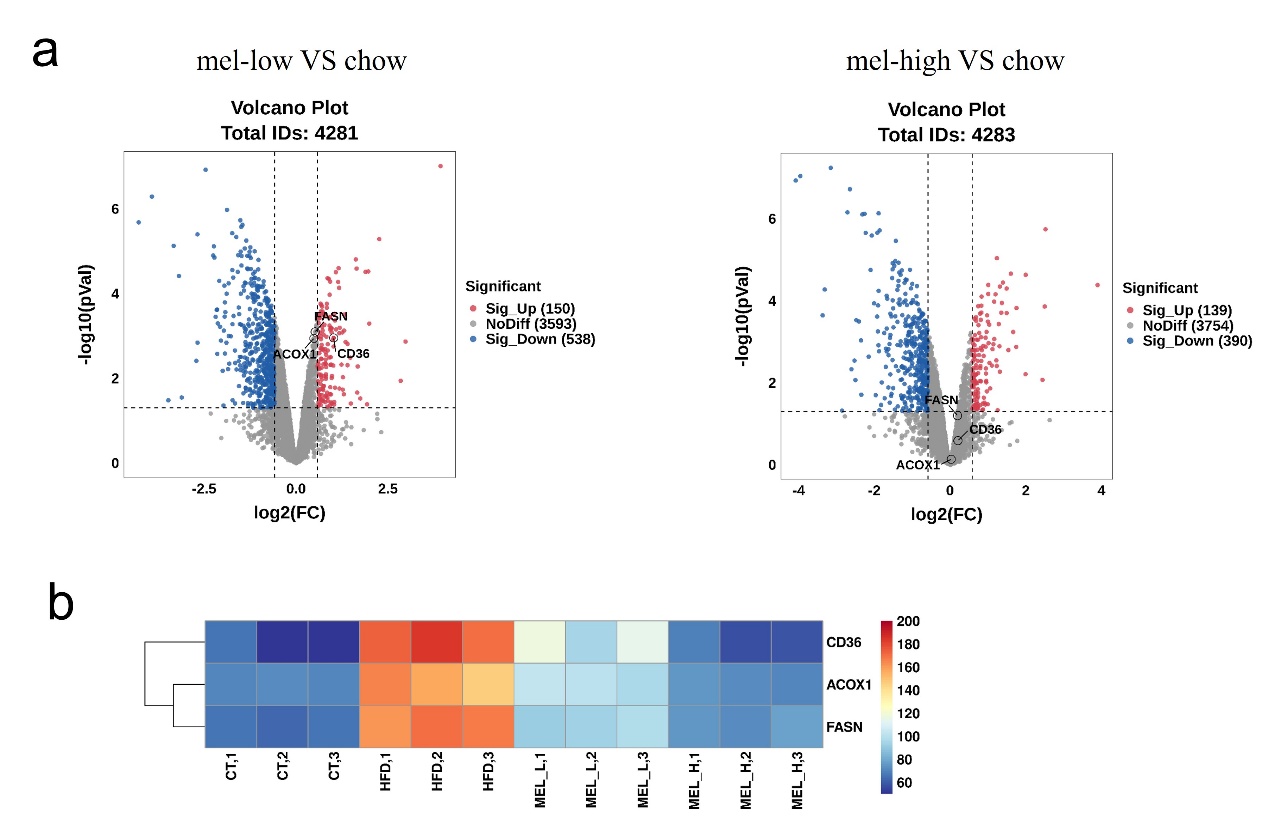


Figure S1. a. Volcano plots depicting the differentially expressed proteins (DEPs) in mouse live cells after different treatments, mel-low vs chow and mel-high vs chow

respectively, n=3. b. Heatmap of CD36, ACOX1, and FASN proteins.

Table S1. The nucleotide sequences of si*Hadha* and siNC

| Name | Sequences (5'-3') |
| --- | --- |
| si*Hadha*#1 | GCUCUCCCAAUCAAUCAAAUUTT |
| si*Hadha*#2 | CCUCUGAUGAAGACGUCCAAUTT |
| si*Hadha*#3 | GCCAAUACAGAAUAGCAACAATT |
| siNC | TTCTCCGAACGTGTCACGT |
